# Randomized clinical trial of omega-3 fatty acid-supplemented enteral nutrition *versus* standard enteral nutrition in patients undergoing oesophagogastric cancer surgery

**DOI:** 10.1002/bjs.7799

**Published:** 2012-01-11

**Authors:** J Sultan, S M Griffin, F Di Franco, J A Kirby, B K Shenton, C J Seal, P Davis, Y K S Viswanath, S R Preston, N Hayes

**Affiliations:** 1 Northern Oesophago-Gastric Cancer Unit, Royal Victoria InfirmaryNewcastle upon Tyne, UK; 2 Institute of Cellular Medicine, Newcastle UniversityNewcastle upon Tyne, UK; 3 Human Nutrition Research Centre, School of Agriculture, Food and Rural Development, Newcastle UniversityNewcastle upon Tyne, UK; 4 Upper Gastrointestinal Surgery, James Cook University HospitalMiddlesbrough, UK

## Abstract

**Background:**

Oesophagogastric cancer surgery is immunosuppressive. This may be modulated by omega-3 fatty acids (O-3FAs). The aim of this study was to assess the effect of perioperative O-3FAs on clinical outcome and immune function after oesophagogastric cancer surgery.

**Methods:**

Patients undergoing subtotal oesophagectomy and total gastrectomy were recruited and allocated randomly to an O-3FA enteral immunoenhancing diet (IED) or standard enteral nutrition (SEN) for 7 days before and after surgery, or to postoperative supplementation alone (control group). Clinical outcome, fatty acid concentrations, and HLA-DR expression on monocytes and activated T lymphocytes were determined before and after operation.

**Results:**

Of 221 patients recruited, 26 were excluded. Groups (IED, 66; SEN, 63; control, 66) were matched for age, malnutrition and co-morbidity. There were no differences in morbidity (*P* = 0·646), mortality (*P* = 1·000) or hospital stay (*P* = 0·701) between the groups. O-3FA concentrations were higher in the IED group after supplementation (*P* < 0·001). The ratio of omega-6 fatty acid to O-3FA was 1·9:1, 4·1:1 and 4·8:1 on the day before surgery in the IED, SEN and control groups (*P* < 0·001). There were no differences between the groups in HLA-DR expression in either monocytes (*P* = 0·538) or activated T lymphocytes (*P* = 0·204).

**Conclusion:**

Despite a significant increase in plasma concentrations of O-3FA, immunonutrition with O-3FA did not affect overall HLA-DR expression on leucocytes or clinical outcome following oesophagogastric cancer surgery. Registration number: ISRCTN43730758 (http://www.controlled-trials.com). Copyright © 2012 British Journal of Surgery Society Ltd. Published by John Wiley & Sons, Ltd.

## Introduction

Radical surgery offers patients with oesophagogastric cancer the best prospect of cure, but perioperative risks are high^1^. Morbidity rates of up to 43 and 60 per cent following oesophageal and gastric cancer surgery respectively have been reported in the UK^2^. Supplementation with omega-3 fatty acids (O-3FAs) has been shown to be beneficial in critically ill patients with acute respiratory distress syndrome and in patients undergoing major abdominal surgery^3^, ^4^.

An immunoenhancing diet (IED) can modulate both the hyperinflammatory and compensatory phases associated with surgery^5^. O-3FAs, especially eicosapentaenoic acid (EPA) and docosahexaenoic acid (DHA), are important constituents of immunonutrition owing to their effects on eicosanoid balance. O-3FAs have anti-inflammatory properties, leading to the production of eicosanoids that are less inflammatory than those produced by omega-6 fatty acids (O-6FAs)^6^.

The expression of HLA-DR is crucial in the specific immune response to infection. Reduced HLA-DR expression correlates directly with infectious complications and continued sepsis^7^, ^8^. HLA-DR expression is reduced on the T lymphocytes of patients who develop postoperative infections^9^.

Existing results of immunonutrition in patients with gastrointestinal cancer are inconsistent. Some have reported reduced infective complications and shortened hospital stays^10^–^16^, whereas others have found no advantages^17^–^19^. These studies are confounded by heterogeneous groups of patients with cancer, numerous centres recruiting small numbers of patients and failure to analyse on an intention-to-treat basis^12^–^14^.

The primary aim of the present prospective randomized trial was to study the effect of perioperative enteral immunonutrition with O-3FAs on clinical outcome in a homogeneous group of patients with oesophagogastric cancer. A secondary aim was to examine the immunological effects of O-3FAs on these patients.

## Methods

Ethical approval was given by the Joint Ethics Committee of Newcastle University and North Tyneside Health Authority, and the Multi-Research Ethics Committee, South Tees Healthcare Trust. Patients eligible for the study had histologically proven oesophageal or gastric malignancy deemed suitable for subtotal oesophagectomy or total gastrectomy with curative intent by the multi- disciplinary team. Eligible patients were also offered access to neoadjuvant chemotherapy within the Medical Research Council (MRC) OE02 (ISRCTN 43 987 580) and ST02 (MRC Adjuvant Gastric Infusional Chemotherapy (MAGIC); ISRCTN 93 793 971) trials.

Patients were randomized in equal numbers into three groups using computer-generated block randomization (http://www.randomization.com) with stratification only for malnutrition. An IED group received Oxepa® (Abbott Nutrition, Maidenhead, UK), a balanced liquid feed enriched with O-3FAs (EPA 0·51 g per 100 ml; DHA 0·22 g per 100 ml) for 7 days before and after operation (1·5 kcal/ml with 6·25 g per 100 ml protein and no free arginine or glutamine). A standard enteral nutrition (SEN) group received Ensure Plus® (Abbott Nutrition), an enteral feed (1·5 kcal/ml with 6·25 g per 100 ml protein) without immunonutrients for 7 days before and after surgery. A control group had no preoperative nutritional support but received enteral Osmolite® (Abbott Nutrition) after surgery, according to clinical requirements and consultant preference. This was a balanced, isotonic liquid feed without immunonutrients and lower energy (1 kcal/ml) and protein (4 g per 100 ml) content.

Double-blinding was used to minimize bias. The SEN and IED feeds were identical in colour and type of container used. An individual not involved in the clinical study labelled the feeds with study numbers. The randomization code was broken after completion of the data and laboratory analysis. Because the control group received no preoperative supplementation, blinding was not possible in this group. All patients underwent a standard nutritional assessment 7 days and 1 day before surgery, 7 days after operation and on discharge. This assessment included measurement of weight, body mass index (BMI), percentage unintentional weight loss over the 3 months before surgery (where malnutrition was defined as greater than 10 per cent bodyweight loss), dietary assessment using 24-h recall and anthropometric measurements. Malnutrition was calculated using the following formula: percentage weight loss = (weight 3 months before surgery − weight at surgery) × 100/weight 3 months before surgery. If weight 3 months before surgery was not documented, weight recalled by the patient was used, or the history was used to inform the dietician of estimated weight loss. Anthropometric measurements (in millimetres) included triceps skinfold thickness (TSF) and mid-arm circumference (MAC), measured by tape measure midway between the acromid process and the olecranon process of the non-dominant arm, with the patient's arm relaxed and hanging downwards. Mid-arm muscle circumference (MAMC) was calculated as MAC − [π× TSF].

All patients underwent standardized surgical resection for upper gastrointestinal cancer, with prophylactic cefuroxime and metronidazole at induction and two post- operative doses. During surgery, either a nasojejunal tube (10/12-Fr; CORPAK MedSystems, Wheeling, Illinois, USA) or feeding jejunostomy tube (Freka® 9-Fr, Fresenius Kabi, Germany; or silicone Foley 12-Fr, Coloplast, Humlebaek, Denmark) was inserted into the proximal jejunum depending on consultant preference. After surgery, routine blood investigations (full blood count, urea and electrolytes, liver function tests and C-reactive protein) were measured 1 and 7 days both before and after operation. Fresh blood samples were centrifuged into plasma, red blood cells and lymphocytes then stored at − 80 °C pending analysis.

Before surgery, all patients were advised to follow their current eating plan and tube feeding was undertaken only in those who were malnourished and unable to manage sufficient oral intake. Supplements were considered an addition and not a replacement for normal oral intake. Supplementation was performed as outpatient therapy for 7 days before surgery in the IED and SEN groups. Preoperative supplementation was set at a fixed rate for all patients to provide an additional 1000 kcal/day, resulting in a daily desired volume of supplemental enteral nutrition of 675 ml. The amount of enteral feed consumed was recorded in a diary.

Immediately after operation, feeding tubes were flushed with water. On the second day, feeding was commenced at 25 ml/h, increased by the third day to 50 ml/h, reaching the desired maximum rate at some point that day. The maximum rate was calculated for each patient according to the predicted protein and calories that the individual was thought to require based on Schofield equations for estimating basal metabolic rate, adjusting for stress or weight loss, and adding a combined factor for activity and diet-induced thermogenesis^20^. Feed was continued at the maximum rate between 4 and 7 days after surgery^12^, ^14^, ^15^. If further feed was required on day 8 because of complications, a need for overnight feeding or for those not managing sufficient oral intake before discharge, the enteral feed was changed to Osmolite®.

The primary endpoint was numbers of infective complications (including use of therapeutic antibiotics), with definitions as published previously^21^. Secondary endpoints were other clinical outcomes, including morbidity, jejuno- stomy and feeding-related complications, length of hospital and critical care stay, and in-hospital mortality, along with plasma concentrations of fatty acids, nutritional status and immunological markers.

Fatty acids were extracted from plasma and lymphocytes, using a modified version of a method described previously^22^, in a planned substudy with recruitment of consecutive patients between September 2005 and September 2006. Fatty acid methyl esters in samples were analysed on a GC-2014 gas chromatograph (Shimadzu, Tokyo, Japan) using helium as the carrier gas and a 30-m BPX70 capillary column (SGE Europe, Milton Keynes, UK). Sample fatty acids were identified using external lipid standards, and concentrations were determined with reference to the internal standard (C21 fatty acid) concentration, expressed on a mass basis for each sample.

HLA-DR expression on monocytes and stimulated T lymphocytes from freshly prepared venous blood was determined by flow cytometry^23^, again as a planned substudy with recruitment of patients between April 2003 and April 2005. HLA-DR expression on monocytes was used to estimate their capacity to present an antigen, and expression on activated T lymphocytes to estimate T lymphocyte activation. Fresh blood was incubated with monoclonal antibodies peridinin chlorophyll-A protein-labelled anti-CD45 (Becton-Dickinson; Franklin Lakes, New Jersey, USA), FITC-labelled anti-CD14 (Becton-Dickinson), FITC-labelled anti-CD3 (Dako, Ely, UK) and phycoerythrin-labelled anti-HLA-DR (Becton-Dickinson) for 20 min at room temperature. Following incubation, erythrocytes were mixed with fluorescence-activated cell sorting lysing solution. The samples were incubated again for 10 min at room temperature. T lymphocytes were activated after stimulation *in vitro* with phorbol myristate acetate for 48 h.

Flow cytometric analysis of HLA-DR expression on monocytes and activated T lymphocytes was carried out by gating the leucocyte population of interest. HLA-DR expression on monocytes was measured in molecules of equivalent soluble fluorochrome. The percentage of activated T lymphocytes expressing HLA-DR was also measured. Measurements were performed 7 days and 1 day before, and 7 days after surgery. The T lymphocyte stimulation index (SI) was defined as the ratio between stimulated and non-stimulated T lymphocytes.

### Statistical analysis

The postoperative infective/septic complication rate after elective oesophagectomy and total gastrectomy at the Northern Oesophago-Gastric Cancer Unit was 50 per cent at the time of study design based on audit figures. A reduction of the rate to 25 per cent was considered clinically significant. Assuming a type I error rate of 0·05, 65 subjects were needed in each arm of the trial to detect a difference of this magnitude with 80 per cent power. Considering a 10 per cent rate of attrition, 73 patients per group were required for the study.

Data were analysed on an intention-to-treat basis with SPSS® for Windows® release 12.0 software (SPSS, Chicago, Illinois, USA). χ^2^ analysis or Fisher's exact test was used for comparison of categorical data. Normally distributed continuous data were analysed by means of one-way ANOVA and repeated measures analysis (Tukey *post hoc* analysis). Kruskal–Wallis *H* test was used for analysis of non-normally distributed data. Differences were considered significant at *P* < 0·050.

## Results

**Fig. 1:**
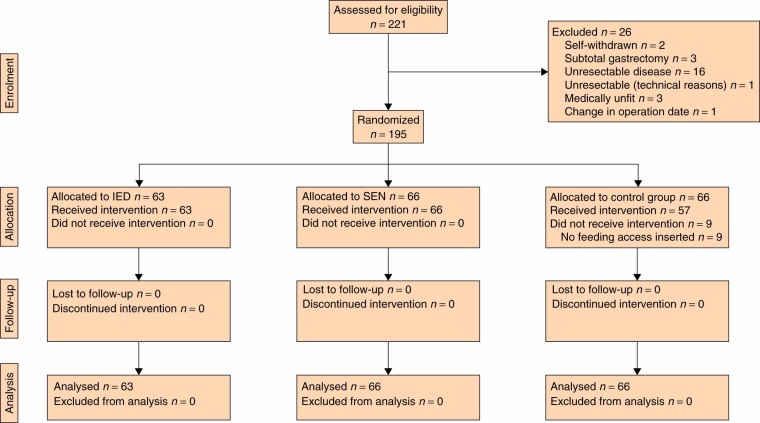
CONSORT diagram for the trial; IED, immunoenhancing diet; SEN, standard enteral nutrition

Some 221 patients were eligible and randomized between April 2003 and January 2007, of whom 26 were excluded (*Fig.* *1*). Data from the remaining 195 patients were used in the analysis. The three trial groups were comparable in terms of age, sex, BMI, recruiting hospital, preoperative staging as node-positive disease, type of operation, malnutrition, median blood loss and proportion requiring blood transfusions (*Table* *1*). Nine patients in the control group did not have feeding access inserted at the time of operation, but there with no differences in the use of nasojejunal tubes (30) or feeding jejunostomy (156) between the groups. Preoperative tube feeding was required in only four patients, with 175 of 195 patients received enteral feeding for more than 8 days after surgery.

**Table 1:** Demographic, surgical and nutritional details

	IED (*n* = 66)	SEN (*n* = 63)	Control (*n* = 66)	P**
Age (years)*	67 (42–79)	60 (42–79)	66 (43–84)	0·060††
Sex ratio (M:F)	50:16	45:18	50:16	0·857
Neoadjuvant chemotherapy	37 (56)	36 (57)	34 (52)	0·816
Malnutrition	8 (12)	3 (5)	5 (8)	0·334
Body mass index (kg/m^2^)†	25·8(5·2)	26·7(3·8)	25·9(4·0)	0·411‡‡
Preop. node-positive disease	44 (67)	46 (73)	41 (62)	0·281
Median ASA grade	II	II	II	0·614
Operation				0·648
Subtotal oesophagectomy	52 (79)	50 (79)	56 (85)	
Total gastrectomy	14 (21)	13 (21)	10 (15)	
Duration of operation (min)†	343(96)	338(90)	363(71)	0·230‡‡
Blood loss (ml)*	675 (150–1774)	620 (150–2875)	633 (160–7640)	0·804††
Total blood transfusion (units)*	0 (0–15)	0 (0–17)	0 (0–18)	0·725††
Patients transfused	13 (20)	15 (24)	13 (20)	0·727
Nutritional information				
Preop. enteral feed volume (ml)†‡	4256(1046)	4264(1046)	—	0·843‡‡
Postop. enteral feed volume (ml)†§	5616(2519)	5150(2334)	5112(3034)	0·528‡‡
Predicted protein (g per kg per day)†¶	1·20(0·15)	1·21(0·12)	1·21(0·12)	—
Predicted energy (kcal per kg per day)†¶	29·5(4·3)	29·7(4·0)	30·0(4·0)	—
Actual protein received (g per kg per day)†	0·72(0·33)	0·62(0·27)	0·55(0·21)	0·001‡‡
Actual energy received (kcal per kg per day)†	17·6(9·3)	14·8(6·6)	13·5(4·9)	0·001‡‡
Maximum feed rate reached#	36 (55)	40 (63)	15 (22)	0·001
Postop. time at maximum feed rate (days)*	3 (1–5)	2 (1–6)	3 (1–3)	0·715††

Values in parentheses are percentages unless indicated otherwise; values are

* median (range) and

† mean(s.d.).

‡ Overall volume of supplementary enteral feed consumed over the 7 days before surgery.

§ Overall enteral nutrition consumed over the 7 days after surgery.

¶ Predicted protein and energy required calculated by the dietician; this represented what the individual was thought to require based on Schofield equations for estimating basal metabolic rate, adjusting for stress or weight loss, and adding a combined factor for activity and diet-induced thermogenesis.

# Patients who achieved a target maximum hourly rate of feed by day 4 after surgery. IED, immunoenhancing diet; SEN, standard enteral nutrition; ASA, American Society of Anesthesiologists.

** χ^2^ analysis (Fisher's exact test if cell < 5), except

†† Kruskal–Wallis H non-parametric test and

‡‡ one-way ANOVA.

There was no difference between IED and SEN groups in the volume of feed received either before or after operation (*Table* *1*), although the proportion of patients who achieved the desired maximum hourly feed was only 55 and 63 per cent in the IED and SEN groups respectively. There were no differences in the median number of days on which patients received the feed at the maximum hourly rate (median 3, 2 and 3 days in the IED, SEN and control groups respectively; *P* = 0·715). The predicted mean(s.d.) target 7-day postoperative volumes calculated for the IED, SEN and control groups were 10 482(1471), 10 306(1344) and 15 351(2117) ml respectively. The number of patients who actually received these target volumes were 9, 11 and 0 respectively (*P* = 0·003), with only 10·3 per cent of patients reaching this target overall. The control group received significantly less protein and energy than the other two groups because of the lower energy and protein contents of this feed. Patients in the control group were less likely to reach the desired maximum feed rate, with only 22 per cent achieving this (*Table* *1*). Owing to the significantly higher target volumes set, patients in the control group failed to reach their maximum targets.

There were no differences between groups in the total number of infective complications, proportion of patients who developed an infective complication, those requiring therapeutic antibiotics, other complications, critical care stay, hospital stay or mortality rate (*Table* *2*). About half of the patients in each group developed an infective complication, and overall there were no differences in the timing of complications or when therapeutic antibiotics were commenced between the three groups. The relative risk (RR) of developing infective complications between the groups, with 95 per cent confidence interval (c.i.), was: SEN *versus* control, 1·06 (0·76 to 1·50); IED *versus* control, 1·08 (0·77 to 1·52); SEN and IED *versus* control, 1·07 (0·79 to 1·45).

**Table 2:** Infective complications, morbidity and mortality

	IED (*n* = 66)	SEN (*n* = 63)	Control (*n* = 66)	*P*†
Infective complications				
Wound infection	9	9	10	1·000
Urinary tract infection	12	7	16	0·156
Respiratory tract infection	20	23	16	0·333
Intra-abdominal abscess	1	0	0	1·000
Feeding tube infection	2	1	0	0·542
Infective diarrhoea	3	2	1	0·698
Septicaemia	3	2	0	0·286
Anastomotic leak	4	7	5	0·578
Total no. of infections	54	51	48	0·854‡
No. of patients with an infective complication	33 (50)	34 (54)	32 (48)	0·817
Critical care stay (days)*	2 (0–75)	4 (0–34)	2 (0–33)	0·569§
Hospital stay (days)*	18 (4–141)	16 (11–116)	16 (11–34)	0·701§
Overall morbidity	43 (65)	37 (59)	38 (58)	0·646
Death	2 (3)	2 (3)	2 (3)	1·000

Values in parentheses are percentages unless indicated otherwise; values are

* median (range).

IED, immunoenhancing diet; SEN, standard enteral nutrition.

† χ^2^ analysis (Fisher's exact test if cell < 5), except

‡ one-way ANOVA and

§ Kruskal–Wallis H non-parametric test.

Changes in anthropometric measurements, including bodyweight, TSF and MAMC, were comparable (*Tables S1* and *S2*, supporting information).

Plasma fatty acid profiles, determined in 54 consecutive patients, are shown in *Table* *3*. The type of feed used had significant influences on arachidonic acid, linoleic acid, EPA and DHA concentrations, and O-6FA to O-3FA ratios over the four time points. The mean ratio of O-6FA to O-3FA before any supplementation was 4·5:1. This fell significantly by 57 per cent in the IED group as the concentration of O-3FA rose significantly following preoperative consumption of the supplement. After surgery, fatty acid concentrations were lower than preoperative values. Postoperative concentrations of EPA were significantly higher in the IED compared with the SEN (*P* = 0·001, Tukey's test) and control (*P* = 0·001) groups. Similar trends were found for DHA; there were significantly higher postoperative concentrations in the IED group than in the SEN (*P* = 0·001) and control (*P* = 0·007) groups.

**Table 3:** Plasma fatty acids and C-reactive protein levels

Fatty acid	Feed	7 days preop.	1 day preop.	1 day postop.	7 days postop.	P*
Linoleic acid (mg/g)	IED	0·71(0·18)	0·57(0·16)	0·30(0·10)	0·40(0·11)	0·019
	SEN	0·68(0·17)	0·65(0·22)	0·33(0·13)	0·46(0·11)	
	Control	0·79(0·22)	0·84(0·26)	0·37(0·09)	0·50(0·14)	
α-Linolenic acid (mg/g)	IED	0·02(0·12)	0·02(0·12)	0·01(0·00)	0·014(0·01)	0·281
	SEN	0·02(0·01)	0·02(0·01)	0·01(0·00)	0·013(0·01)	
	Control	0·03(0·02)	0·02(0·02)	0·01(0·00)	0·013(0·01)	
Arachidonic acid (mg/g)	IED	0·31(0·07)	0·29(0·87)	0·18(0·59)	0·21(0·54)	0·018
	SEN	0·30(0·68)	0·28(0·94)	0·15(0·60)	0·20(0·69)	
	Control	0·32(0·13)	0·38(0·11)	0·20(0·06)	0·24(0·76)	
EPA (mg/g)	IED	0·05 0·23)	0·28(0·14)	0·09(0·05)	0·21(0·13)	< 0·001
	SEN	0·05(0·04)	0·06(0·07)	0·02(0·02)	0·03(0·04)	
	Control	0·07(0·06)	0·06(0·02)	0·02(0·01)	0·03(0·02)	
DHA (mg/g)	IED	0·18(0·05)	0·24(0·09)	0·15(0·04)	0·17(0·05)	0·007
	SEN	0·18(0·08)	0·17(0·08)	0·10(0·05)	0·11(0·06)	
	Control	0·18(0·05)	0·19(0·05)	0·11(0·06)	0·13(0·40)	
O-6FA:O-3FA ratio	IED	4·52(1·57)	1·94(1·25)	2·11(0·95)	1·90(1·03)	< 0·001
	SEN	4·45(1·42)	4·13(1·22)	4·07(1·31)	5·01(1·70)	
	Control	4·52(1·77)	4·82(1·37)	5·19(3·12)	4·75(1·13)	
CRP (mg/l)	IED	10·3(14·1)	7·0(5·1)	98·0(49·7)	95·6(85·6)	0·673
	SEN	11·8(14·1)	7·6(5·4)	130·2(61·0)	102·0(53·1)	
	Control	6·8(6·8)	5·7(2·6)	126·4(89·7)	96·7(67·6)	

Values are mean(s.d.). IED, immunoenhancing diet; SEN, standard enteral nutrition; EPA, eicosapentaenoic acid; DHA, docosahexaenoic acid; O-6FA, omega-6 fatty acid; O-3FA, omega-3 fatty acid; CRP, C-reactive protein.

* Repeated measures analysis; tests of between-subjects effects (to assess whether there were any significant differences caused by the three types of feed).

Lymphocyte fatty acid profiles in the same patients are shown in *Table* *4*. Lymphocyte EPA and DHA concentrations were significantly higher once treatment had been started in the IED group compared with the SEN and control groups.

**Table 4:** Lymphocyte fatty acid concentrations

Fatty acid	Feed	7 days preop.	1 day preop.	1 day postop.	7 days postop.	P*
Linoleic acid (mg/g)	IED	4·39(1·11)	4·41(1·00)	4·14(1·31)	4·77(0·75)	0·023
	SEN	3·39(1·14)	4·34(0·57)	3·95(1·04)	4·39(1·19)	
	Control	4·54(1·34)	4·75(1·37)	4·88(1·67)	4·51(1·13)	
α-Linolenic acid (mg/g)	IED	1·10(1·14)	0·70(0·54)	1·08(1·11)	0·56(0·46)	0·394
	SEN	0·76(0·61)	0·70(0·62)	1·31(1·08)	0·71(0·64)	
	Control	0·67(0·60)	0·80(0·72)	0·97(1·21)	0·41(0·41)	
Arachidonic acid (mg/g)	IED	1·08(0·32)	1·07(0·25)	0·85(0·32)	0·95(0·21)	0·378
	SEN	1·02(0·39)	1·10(0·34)	0·95(0·88)	0·92(0·35)	
	Control	1·12(0·31)	1·10(0·37)	1·05(0·52)	1·02(0·39)	
EPA (mg/g)	IED	0·40(0·44)	1·76(0·88)	1·12(0·66)	1·78(0·88)	< 0·001
	SEN	0·26(0·15)	0·25(0·18)	0·16(0·33)	0·23(0·23)	
	Control	0·39(0·33)	0·38(0·47)	0·25(0·29)	0·22(0·14)	
DHA (mg/g)	IED	1·51(0·40)	1·81(0·43)	1·51(0·43)	1·83(0·43)	< 0·001
	SEN	1·38(0·20)	1·43(0·30)	1·34(0·32)	1·45(0·29)	
	Control	1·64(0·51)	1·58(0·54)	1·38(0·38)	1·49(0·44)	

Values are mean(s.d.). IED, immunoenhancing diet; SEN, standard enteral nutrition; EPA, eicosapentaenoic acid; DHA, docosahexaenoic acid.

* Repeated measures analysis; tests of between-subjects effects (to assess whether there were any significant differences caused by the three types of feed).

HLA-DR expression on monocytes and T lymphocytes was measured in 45 consecutive patients (IED, 16; SEN, 14; control, 15). There was a significant increase in HLA-DR expression on monocytes between 7 days and 1 day before operation in the IED group (*P* = 0·001) (*Table* *5*). Despite the overall effects of surgery on HLA-DR expression on monocytes through the perioperative period (*P* = 0·001), there were no differences between the groups at the various time points (*P* = 0·538).

**Table 5:** HLA-DR expression on monocytes and T lymphocytes

	IED	SEN	Control	P*
Monocytes (MESF)				0·538
7 days preop.	18 109(7017)	26 853(11 719)	23 588(10 666)	
1 day preop.	27 995(18 621)	25 154(12 939)	20 756(9371)	
7 days postop.	10 555(8137)	13 605(8216)	17 679(14 161)	
Non-stimulated T lymphocytes (%)				0·814
7 days preop.	15·4(7·1)	14·3(6·0)	14·6(6·6)	
1 day preop.	16·2(8·8)	15·9(7·2)	14·6(6·2)	
7 days postop.	16·5(6·2)	19·8(11·2)	16·5(7·4)	
Stimulated T lymphocytes (%)				0·204
7 days preop.	32·3(8·2)	38·1(11·9)	37·3(15·0)	
1 day preop.	37·7(12·7)	36·6(14·5)	35·84(13·5)	
7 days postop.	40·1(10·1)	29·2(12·4)	38·1(16·0)	
T lymphocyte SI				
7 days preop.	2·32(0·78)	2·75(0·81)	2·71(1·02)	
1 day preop.	2·44(0·82)	2·44(0·95)	2·34(0·87)	0·814
7 days postop.	2·84(1·69)	1·71(0·94)	2·56(0·56)	

Values are mean(s.d.). IED, immunoenhancing diet; SEN, standard enteral nutrition; MESF, molecules of equivalent soluble fluorochrome. SI, stimulation index (ratio between stimulated and non-stimulated T lymphocytes).

* Repeated measures analysis; tests of between-subjects effects (to assess whether there were any significant differences caused by the three types of feed).

The percentage of stimulated T lymphocytes expressing HLA-DR was similar for all treatments and there were no significant differences between the groups at any time point (*P* = 0·204). Similarly, there was no significant difference in the T lymphocyte SI among the three groups throughout the perioperative period (*P* = 0·814).

## Discussion

A strength of the present study, compared with previous immunonutrition trials involving patients undergoing cancer surgery, was the relative homogeneity of the patient group. Only those receiving standardized resections for oesophagogastric cancer at two tertiary centres were included. During design of the trial, consideration was given to making the control group completely ‘nil by mouth’ with no postoperative supplementation, but this was discounted as ethical problems were foreseen because published data have shown benefit from postoperative enteral feeding^23^. Some of the variability in the results obtained seems likely to reflect problems in patient compliance both before and after operation. Before surgery, patients reported a dislike of the supplement regarding taste, bloating and/or nausea. After the procedure, overall only 46·7 per cent of the patients reached the maximum planned feeding rate owing to problems with tolerance and/or complications, including diarrhoea, ileus, nausea, vomiting or bloating. These factors limit clinical data interpretation but nevertheless reflect clinical practice.

Interpretation of the anthropometric data may be limited by inter-rater variability despite attempts to create standards. In this study, all measurements were made by two specialist dieticians. The coefficient of variation has been reported as 4·7 per cent for arm circumference, with a difference of at least 2·68 cm needed to demonstrate a true change^24^.

Despite problems with feeding, there were clear differences in circulating O-3FA concentrations in the plasma of patients in the IED group, indicating that this component of the supplement was assimilated successfully. In a previous study small increases in O-3FA intake, above the adequate daily intake of 0·65 g EPA and DHA, resulted in a rapid change in the fatty acid composition of blood cell membranes, with an enrichment of O-3FA^25^. Supplementation with 1 g/day O-3FA increased total O-3FA by 62 per cent within 1 week and this remained stable for 12 weeks^25^. Although the maximum feeding rate was not achieved in all patients in the present study, because the EPA content of the supplement was high, patients in the IED group on average received much higher amounts of O-3FA before and after surgery (6 and 8 g/day EPA respectively). Resulting changes in O-3FA concentrations and the O-6FA to O-3FA ratio were greater than those reported previously^25^, ^26^.

Critical concentrations of plasma and lymphocyte O-3FAs required for a clinical response are unknown, but the intervention in this study had a significant impact on fatty acid concentrations. Regarding surgical patients, two small studies demonstrated that EPA and DHA are associated with significantly increased mitogen-stimulated lymphocyte proliferation^8^, and reported significantly reduced HLA-DR expression on monocytes of patients who did not receive O-3FA supplementation compared with those who did^4^.

In the present study, immunonutrition before and after surgery conferred no advantage in overall clinical outcome compared with an isocaloric, isonitrogenous standard enteral feed, or postoperative enteral feed alone with a lower energy and protein content. The number of infective complications, and the use and duration of antibiotics, was similar for all groups. The lack of effect of O-3FA on infective complications was supported by the immunological results. Perioperative administration of IED was associated with increased HLA-DR expression on monocytes between days 7 and 1 before operation. It is not clear whether the rise in HLA-DR in the IED group seen 1 day before the procedure was due to the low values measured for this group at 7 days before surgery or reflects O-3FA supplementation. The magnitude of the postoperative fall in HLA-DR expression from the value measured 7 days before operation was similar for both IED and SEN. These findings seem unlikely to be important from an immunological point of view. There were no significant differences in HLA-DR expression on monocytes among the three groups throughout the whole perioperative period. As demonstrated by the measurement of HLA-DR expression on stimulated T lymphocytes and the T lymphocyte SI, the ability of monocytes to activate T lymphocytes in the postoperative period was preserved in all groups.

Blood transfusions are known to have immunological effects. Natural killer cell function is significantly impaired for up to 30 days after surgery in patients transfused with whole blood^27^. In the present study, 41 of 195 patients required blood transfusion during their hospital admission, with no difference in distribution between study groups.

Infective complication rates have been shown to be reduced in patients receiving perioperative IED^13^, ^16^, but in those studies the data were not analysed on an intention-to-treat basis. A reanalysis of the present study based on treatment given, in terms of patients reaching their preoperative target volume or maximum postoperative hourly feed rate, showed no difference in outcome (data not shown). Two other studies have also failed to demonstrate a reduction in infective complications with perioperative IED^17^, ^18^ and a further study showed no benefit from IED administered after operation in patients undergoing surgery for upper gastrointestinal malignancies^19^. A meta-analysis that included 22 randomized trials of immunonutrition in postoperative and critically ill patients did imply a significant reduction in infective complications (RR 0·66, 95 per cent c.i. 0·54 to 0·80) but with no improvement in mortality (RR 1·10, 0·93 to 1·13)^28^. Of 18 studies with data on infectious complications, ten had confidence intervals that included 1·0 and four showed no benefit. Effects appeared to be greater, however, in postoperative rather than critically ill patients, indicating that characterization of patients is important when reporting and comparing studies. A meta-analysis of 21 randomized trials involving major gastrointestinal surgery also concluded that immunonutrition decreased morbidity and hospital stay, but not mortality rates^29^. However, the wide disparity in results from more than 30 randomized trials aimed at assessing the clinical significance of immunonutrition in the past two decades limits firm conclusions.

If the clinical results of the present trial are accepted as the basis for a future study, then to achieve a more modest reduction in complication rate of 20 per cent would require a sample size of over 1800 patients. This is considered unachievable, unless undertaken on a multicentre basis.

Current local practice is, therefore, to provide preoperative supplementation only to malnourished patients or those unable to consume food orally because of symptoms or the disease process. The optimal quantity and combination of nutrients, the timing of their delivery and the patient group(s) most likely to benefit from immunonutrition remain uncertain.

## Supporting information

**Table S1** Mean bodyweight during the study (Word document)

**Table S2** Triceps skinfold thickness and mid-arm muscle circumference during the study (Word document)

Please note: John Wiley & Sons Ltd is not responsible for the functionality of any supporting materials supplied by the authors. Any queries (other than missing material) should be directed to the corresponding author for the article.
