# Supplementary material for: Randomized clinical trial of omega-3 fatty acid-supplemented enteral nutrition versus standard enteral nutrition in patients undergoing oesophagogastric cancer surgery
Source: Br J Surg. 2012 Jan 11;99(3):346–55. doi: 10.1002/bjs.7799 (PMC3625735; doi:10.1002/bjs.7799)
Supplement: Supplementary file 1 [file bjs0099-0346-SD1.doc]

**BJS7796**

### Randomized clinical trial of omega-3 fatty acid-supplemented enteral nutrition *versus* standard enteral nutrition in patients undergoing oesophagogastric cancer surgery

J. Sultan, S. M. Griffin, F. Di Franco, J. A. Kirby, B. K. Shenton, C. J. Seal, P. Davis, Y. K. S. Viswanath, S. R. Preston and N. Hayes

**Table S1** Mean bodyweight during the study

|  | Weight (kg) | | | | | *P** |
| --- | --- | --- | --- | --- | --- | --- |
|  | 7 days preop. | 1 day preop. | 7 days postop. | On discharge | At follow-up on ward |  |
| IED (*n =* 56) | 78.9(17.6) | 79.9(17.7) | 84.8(17.0) | 78.7(17.5) | 73.8(15.8) | 0.322 |
| SEN (*n =* 48) | 75.2(11.7) | 76.1(11.7) | 80.5(12.9) | 74.2(12.6) | 70.4(12.0) |  |
| Control (*n =* 53) | 75.8(15.3) | 76.0(15.4) | 80.6(15.2) | 74.6(14.9) | 71.5(14.2) |  |
| Mean (*n =* 157) | 76.7(15.2) | 77.4(15.3) | 82.1(15.3) | 75.9(15.3) | 72.0(14.2) | 0.001 |

Values are mean(s.d.). IED, immunoenhancing diet; SEN, standard enteral nutrition. *Repeated measures analysis.

**Table S2** Triceps skinfold thickness and mid-arm muscle circumference during the study

|  | 7 days preop. | 7 days postop. | 14 days postop. | *P** |
| --- | --- | --- | --- | --- |
| Triceps skinfold thickness (mm) |  |  |  |  |
| IED (*n =* 56) | 17.8(7.4) | 17.6(6.4) | 16.7(6.2) | 0.307 |
| SEN (*n =* 50) | 16.7(7.0) | 16.1(6.8) | 15.4(6.3) |
| Control (*n =* 53) | 15.9(6.0) | 16.0(5.9) | 14.9(5.2) |
| Mean (*n =* 159) | 16.8(6.8) | 16.6(6.4) | 15.7(5.9) | 0.001 |
| Mid-arm muscle circumference (cm) |  |  |  |  |
| IED (*n =* 56) | 24.1(3.4) | 23.7(3.5) | 23.1(3.1) | 0.417 |
| SEN (*n =* 50) | 24.2(2.7) | 23.7(2.4) | 23.4(2.4) |
| Control (*n =* 53) | 25.0(3.4) | 24.1(3.0) | 23.8(2.8) |
| Mean (*n =* 159) | 24.4(3.2) | 23.8(3.0) | 23.5(2.8) | 0.001 |

Values are mean(s.d.). IED, immunoenhancing diet; SEN, standard enteral nutrition. *Repeated measures analysis.
